# Study the effect of various wash-coated metal oxides over synthesized carbon nanofibers coated monolith substrates

**DOI:** 10.1371/journal.pone.0219936

**Published:** 2019-07-31

**Authors:** Mohamad Rasool Malekbala, Soroush Soltani, Suraya Abdul Rashid, Luqman Chuah Abdullah, Thomas Shean Yaw Choong

**Affiliations:** 1 Department of Chemical and Environmental Engineering/ Sustainable Process Engineering Research Center (SPERC), Universiti Putra Malaysia, Selangor, Malaysia; 2 Materials Processing and Technology Laboratory, Institute of Advanced Technology, Universiti Putra Malaysia, Selangor, Malaysia; Institute of Materials Science, GERMANY

## Abstract

In this research work, carbon nanofibers (CNFs) were synthesized on honeycomb monolith substrates using injection chemical vapor deposition (ICVD) technique. The effect of various wash-coated materials and catalyst promoter on the growth rate of CNFs on monolith substrates were examined. The characteristics of the synthesized CNFs-coated monolith composites were examined using Raman spectroscopy, Brunauer–Emmett–Teller (BET), thermogravimetric analysis (TGA), field emission scanning electron microscopy (FE-SEM), and Transmission electron microscopy (TEM) techniques. According to the textural characterization study, the specific surface area and pore volume of CNFs-coated monolith composites were significantly improved as compared to bare monolith which might be attributed to the growth of highly pure and aligned CNFs over monolith substrate. Besides that, the synthesized CNFs-coated monolith possessed extremely well thermal stability up to the temperature of 550 °C which was corresponded to the strong attachment of highly graphitized CNFs over monolith substrates.

## 1. Introduction

Over the past few years, advanced carbon-based materials such as carbon nanofibers (CNFs) and carbon nanotubes (CNTs) have been the center of research due to their impressive textural, physicochemical, thermal, electrical, optical, and mechanical characteristics. The combination of these unique and remarkable properties suggests opportunities for wide range of multifunctional applications consisting of film industry, optical fiber communication, optical waveguide, electronic nanodevices, and sensors [1].

The CNFs are generally fabricated in cylindrical or conical structures which the structure highly depends on the arrangements of modified graphene sheets. Besides, the diameter and length of the CNFs can be adjusted according to their desired applications. It is well-known that CNFs can be grown directly on the monolith substrates due to hydrocarbon exposure at elevated temperature. Acid immersion and oxidation in air can be employed to treat the surface prior to hydrocarbon exposure for CNFs growth. However, there is a challenge of how the surface and morphology affect the CNFs growth. It should be noted that the process conditions can significantly improve the growth of CNFs on the monolith and the other industrially relevant substrates. The main distinguishing characteristic of CNFs from CNTs materials is the existence of graphene sheets of different shapes [2]. In general, the CNFs are synthesized through various techniques including arc discharge, laser ablation and variety of chemical vapor deposition (CVD) methods. Among all the mentioned fabrication methods, the CVD technique is highly preferred due to various criteria including high purity, growing control, easy industrial development, and inexpensive synthesis cost. However, some modifications need to be performed in order to increase carbon yield [3].

Catalytic chemical vapor deposition used for CNFs synthesis can be performed in two ways as following: (i) pre-coating a substrate with a metal particle [4], and (ii) depositing catalyst onto the substrate in presence of a carbon source during the reaction called injection chemical (ICVD) method [5,6]. Through the ICVD technique, the pre-preparation step can be avoided; however, there is less control over the size of the resulting nanoparticles which may affect the CNFs properties [7]. Up to now, the ICVD technique has been performed in presence of variety of catalysts, carbon and hydrogen precursors at various temperatures over different substrates [8,9]. Catalyst plays a crucial role in the catalytic growth method. The compatibility of the catalyst components including the active metal, promoter and support is indeed unique. Transition metals, such as Co, Ni, Fe, and etc., have been frequently applied for catalyzing the growth of CNFs, however, the presence of a proper catalyst promoter, such as Fe, Co, Mn, Mg, Al, Ni, Mo, Cu, Pd, Pt, etc., has been effectively recommended [10–12]. A catalyst promoter can indirectly participate in the catalytic growth of CNFs. Moreover, it can either modify the morphology of the active metal to ease the growth of CNFs or contribute in enhancing metal-support interaction of the catalyzing system [13–14].

It is worthy to mention that powder form of CNFs may cause some obstacles through post-coating over substrate such as formation of fines for slurry phase operations and high pressure drop for gas phase operations [15–18]. To overcome these drawbacks, several different substrates have been used such as metallic filters, foams, silica gel beads, carbon felts, and ceramic support. Recently, monolithic materials have attracted a great deal of attention due to their magnificent advantages such as the high void fraction, low pressure drop, thermal shock resistance. On the other hand, cordierite monoliths possess a low specific surface area of 4 m^2^/g which may lower down their capability as a proper substrate [19]. In this regard, monoliths are typically coated with a material possessing high specific surface area in order to highly maximize dispersion of metal particles on the surface [20].

The synthesis of nanofibers on a substrate in presence of a proper catalyst promoter can highly improve their performance for different applications. The aim of using a catalyst promoter is to simultaneously increase specific surface area and eliminate mass-transfer limits between phase because of the hydraulic regime developed within the capillary-channels of the monolith. The principal was to investigate the effect of various coated monolith substrates for growing of CNFs and to compare the growth yield. Here, the CNFs-coated honeycomb monolith substrates were successfully synthesized using ICVD technique. The effect of various wash-coated materials (Iron nitrate, Cobalt nitrate, and Aluminum sulfate) and catalyst promoter on the growth rate of CNFs on monolith substrates were examined. An important point that was taken into consideration was the possibility of the efficient synthesis of CNFs from an eco-friendly precursor. In this regard, furfuryl alcohol was selected as a naturally derived precursor due to its high carbon yield and insignificant toxicity. Moreover, the textural, structural and morphological characteristics and thermal stability of the fabricated CNFs-coated monolith were studied.

## 2. Materials and methods

### 2.1. Chemicals and materials

Cordierite monoliths (2MgO•2Al_2_O•5SiO_2_) substrate with cell density of 400 cells per square inch and the size of 25(L)×20(D) mm^2^ were supplied by Beihai Haihuang Chemical Packing Co.Ltd., China. Iron nitrate [Fe(NO_3_)_3_•9H_2_O], Cobalt nitrate [Co(NO_3_)_3_•9H_2_O], ferrocene [Fe(C_5_H_5_)_2_], Aluminum sulfate hexadecahydrate [Al_2_(SO_4_)_3_•16H_2_O] and furfuryl alcohol [C_5_H_6_O_2_] were purchased from Sigma-Aldrich, Malaysia. Purified Argon (Ar; 99.99%) and Helium (H_2_; 99.99%) gases were provided by Linde AG, Germany. All these analytical-grade chemicals were used as received without further purification.

### 2.2. Wash-coating of monoliths

In order to improve the growth rate of CNFs on the monolith substrate, different coated monolith substrates were initially fabricated. The wash-coating procedure was conducted with 400 cpsi cordierite monolith using Iron nitrate, Cobalt nitrate, and Aluminum sulfate. Initially, the bare monolith was soaked into a solution for 2 h where 10 g of Aluminum sulfate was dissolved in 100 mL distilled water. Afterwards, drying process was taken place in an electric oven at 110 °C for 24 h, followed by calcination at 900 °C for 3 h in an open cap tube furnace reactor with heating rate of 10 °C/min (labeled as S1).

Other wash-coated samples were prepared at the same condition using nitrate salt. These samples were labeled as S2 and S3 for cobalt nitrate and iron nitrate, respectively. Another sample was prepared by coating of iron nitrate on sample S1 at the same condition and labeled as S4.

Decomposition of nitrate salt to metal oxides are given as:

$${\text{Al}}_{2}{\left({\text{SO}}_{4}\right)}_{3}\left(\text{heat}\right)\to {\text{Al}}_{2}{\text{O}}_{3}+3{\text{SO}}_{3}$$

$$2\text{Co}{\left({\text{NO}}_{3}\right)}_{2}\left(\text{heat}\right)\to 2\text{CoO}+4{\text{NO}}_{2}+{\text{O}}_{2}$$

$$4\text{Fe}{\left({\text{NO}}_{3}\right)}_{3}\left(\text{heat}\right)\to 2{\text{Fe}}_{2}{\text{O}}_{3}+12{\text{NO}}_{2}+3{\text{O}}_{2}$$

### 2.3. Synthesis of CNFs-coated monolith

In the current research work, for synthesis of CNFs, the tube furnace reactor was comprised of a 6.2 cm diameter quartz tube and a maximum of 65 cm heating zone. To ensure the elimination of oxygen gas inside the reaction tube, Ar gas was flushing into the tube with flowrate of 10 mL/min. The furnace was heated up to 700 °C and remained at same temperature for 2 hwhile the gas composition was controlling by flow meter. A mixture of furfuryl alcohol/ferrocene along with a mixture of H_2_:Ar with molar ratio of 1:1 was continuously introduced in to the tubular quartz at a flow rate of 0.1 mL/min. As the growth time of 2 h was completed, the furnace was cooled down to room temperature under Ar-flow (10 mL/min). The synthesized CNFs-coated monolith composite was taken for further examination.

### 2.4. Proposed mechanism of the catalytic growth of CNFs

The strategy for the catalytic growth of CNFs can be explained based on vapor-liquid-solid (VLS) model. The catalytic growth of CNFs occurred through multiple steps in accordance with the following reaction steps:

(i) Vaporized ferrocene molecules underwent decomposition in the gas-phase to atomic Iron.
(ii) These Iron atoms bonded together to form Iron nanoclusters and deposited on the surface of the wash-coated monolith.
(iii) Furfuryl alcohol dissociated and then decomposed in the reaction zone.
(iv) Finally, the carbon atoms dissolved in the metallic catalytic nanoparticles, then saturated and precipitated to form CNFs.

Based on vapor-liquid-solid (VLS) model represented by Jiang et al [21], the growth process of CNFs involved the vapor phase precursor, liquid phase carbon-metal solution and solid-phase CNFs. The precursor molecules are in bulk vapor-phase, of which the chemical potential plays as a function of its partial pressure. Throughout growth of CNFs, the partial temperature and pressure are usually stayed constant.

CNFs are in solid-phase, of which the chemical potential is equivalent to the chemical potential of oriented carbon plus additional curvature energy. As a result, if there is no diameter variation during growth of CNFs, the chemical potential stays constant. It should be noted that for the nanoscale catalyst, the initial state is in the solid-state as long as the precursor molecules are not fed in. As the reaction between nanoscale catalyst and precursor molecules accrued, it will be turned into liquid-state carbon-metal solution. As the concentration of carbon increased to supersaturated level, CNFs layer will nucleate over the liquid-phase catalyst.

### 2.5. Characterization of CNFs-coated monolith

The Brunauer-Emmett-Teller (BET) model was employed to study the specific surface areas (SBET) and also total pore volume of the fabricated CNFs-coated monolith substrate by nitrogen (N_2_) adsorption-desorption isotherm methods, using Micromeritics-Tristar apparatus. Raman spectroscopy was introduced to identify chemical bonding of the fabricated CNFs-coated monolith substrates, using a Perkin-Elmer GXFT-IR-Raman spectrometer equipped with a NdYAG laser with the wavelength of 1064 nm. In order to evaluate the weight-losses of the functional groups on the surface of the fabricated CNFs-coated monolith composite, thermogravimetric analysis (TGA, Netzsch-STA-409) was employed. A typical TGA testing was conducted from 26 °C to 800 °C with a heating rate 10 °C/min in oxygen flowrate of 100 mL/min. The morphological characterization of the fabricated CNFs-coated monolith substrate was assessed through using transmission electron microscopy (TEM, Philips-CM-12) and field emission scanning electron microscopy (FE-SEM, Sirion-100, USA). X-ray diffraction (XRD) was utilized to characterize the purity of the CNF coated on monolith, using an XRD-6000, Shimadzu by changing the angle within the range of 15–50°.

Carbon yield of the coated CNFs on the monolith substrate was calculated using Eq (i):

$$\text{CNF}\left(\text{wt}.\%\right)=\frac{m_{t}-m_{i}}{100\times m_{i}}\times 100$$ (i)

Where; mt is the overall mass gained by the end of the process, and mi is the mass of the wash-coated monolith.

## 3. Results

### 3.1. Coating of CNFs on raw and wash-coated monolith substrates

**Fig 1:**
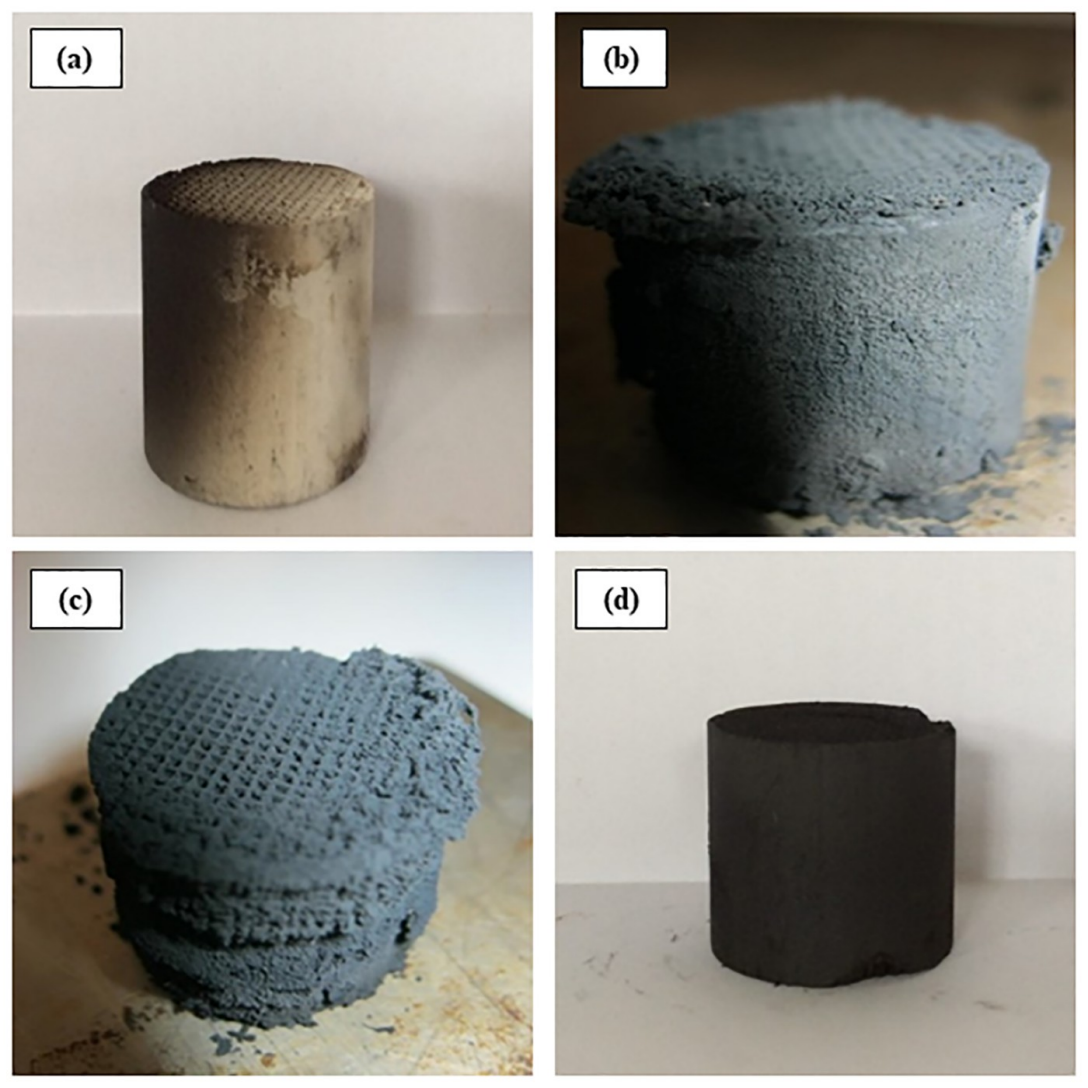
(a) CNFs-coated on raw monolith; (b) CNFs-coated on Al wash-coated monolith; (c) CNFs-coated on Co wash-coated monolith; (d) CNFs-coated on Al/Fe wash-coated monolith.

The CNFs were coated on raw honeycomb monolith substrates using ICVD technique at the following reaction conditions: furfuryl alcohol 9 mL, ferrocene 1 g, and calcination temperature of 700 °C. As it can be seen in Fig 1(a), coating of the CNFs on raw monolith was unsuccessfully occurred. The commercial prepared raw monolith possessed an extremely low specific surface area of 4 m^2^/g and low porosity of 0.19 cm^3^/g. As it was expected, very insignificant number of CNFs deposited on the pore sites and the rest coated over the walls of the monolith. Moreover, it was observed that the growth of CNFs on the monolith substrate preferentially occurred on the quartz tube, rather than on the monolith substrate. In order to maximize the growth rate of CNFs on the monolith substrate, different coated metal monoliths were fabricated.

The bare monolith was wash-coated with alumina sulphate and BET surface areas increased from 1 to 31.2 m^2^/gr. The injection CVD method has resulted in cordierite monoliths with very different properties and behavior for the same growth conditions on the wash-coated cordierite. As shown in Fig 1(b), CNFs coated on Al wash-coated monolith are very disordered, with growth in random orientations, resulting in a highly uneven layer across the wash-coated monolith. This disordered growth previously being reported in different studies [22,23]. The wash-coated samples using other catalyst promotors with the same conditions were investigated. It was observed that samples are uniformly coated and turned the color of monolith from white to black (Fig 1(c) and 1(d)).

### 3.2. Effect of catalyst promoter

The effect of catalyst promoter on the growth rate of CNFs on the monolith substrate was examined while the other reaction conditions were kept constant. Fig 1(d) showed uniformly coated of CNFs on the monolith substrate where the color of monolith turned from white to black.

**Fig 2:**
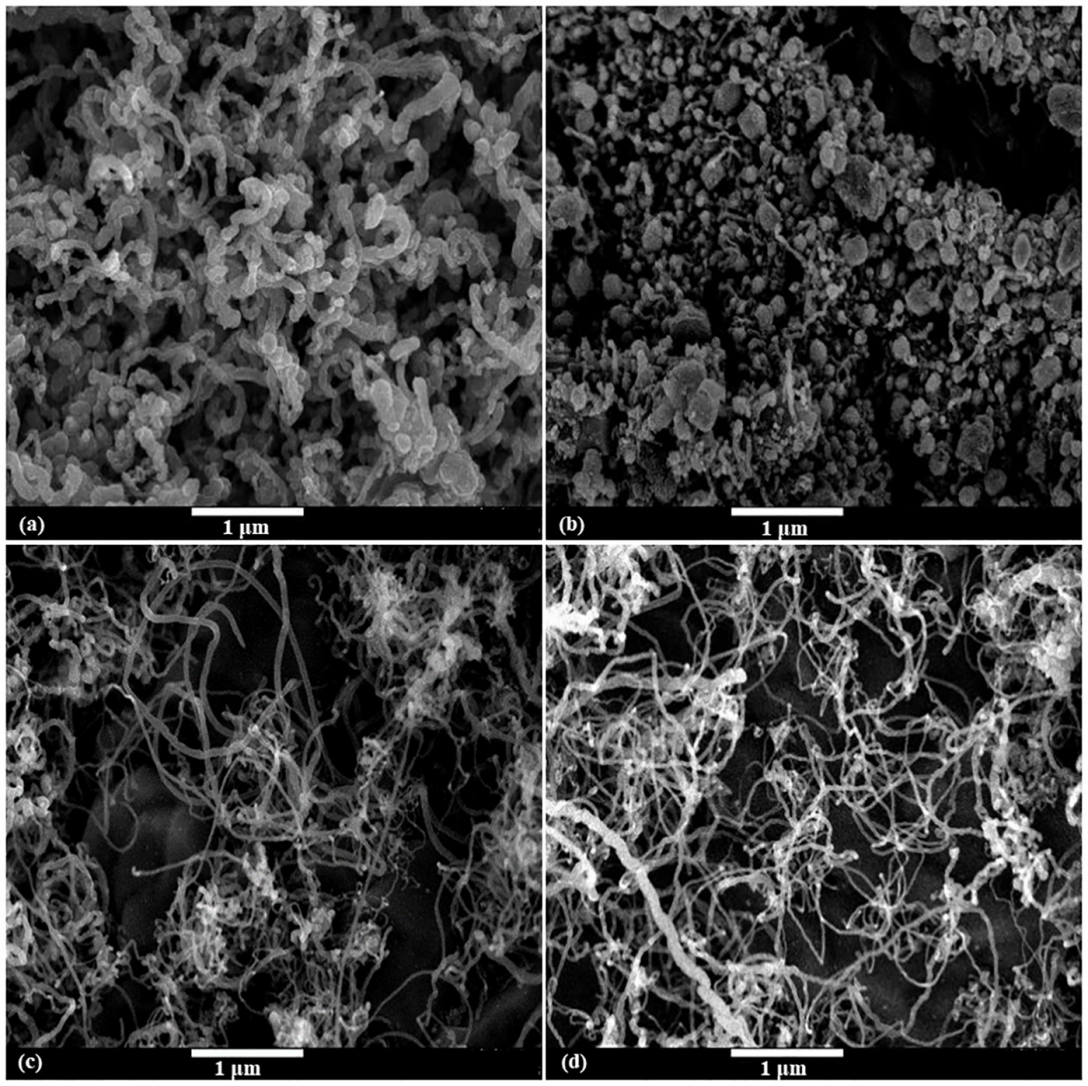
FE-SEM images of different wash-coating samples (a) S1; (b) S2; (c) S3; (d) S4.

In order to assess the presence, quality and structure of the CNFs on monolith substrate, FE-SEM and Raman characterizations were applied. A comparison study was carried out between synthesized CNFs-coated monolith substrate using different wash-coated materials (S1, S2, S3, S4). As it is shown in Fig 2, as cobalt was applied as catalyst promoter (S2), most of the products were either amorphous carbon or carbon-wrapped particles and CNFs could hardly be obtained. However, the formation of CNFs was proven at S1, S3, and S4 samples.

**Fig 3:**
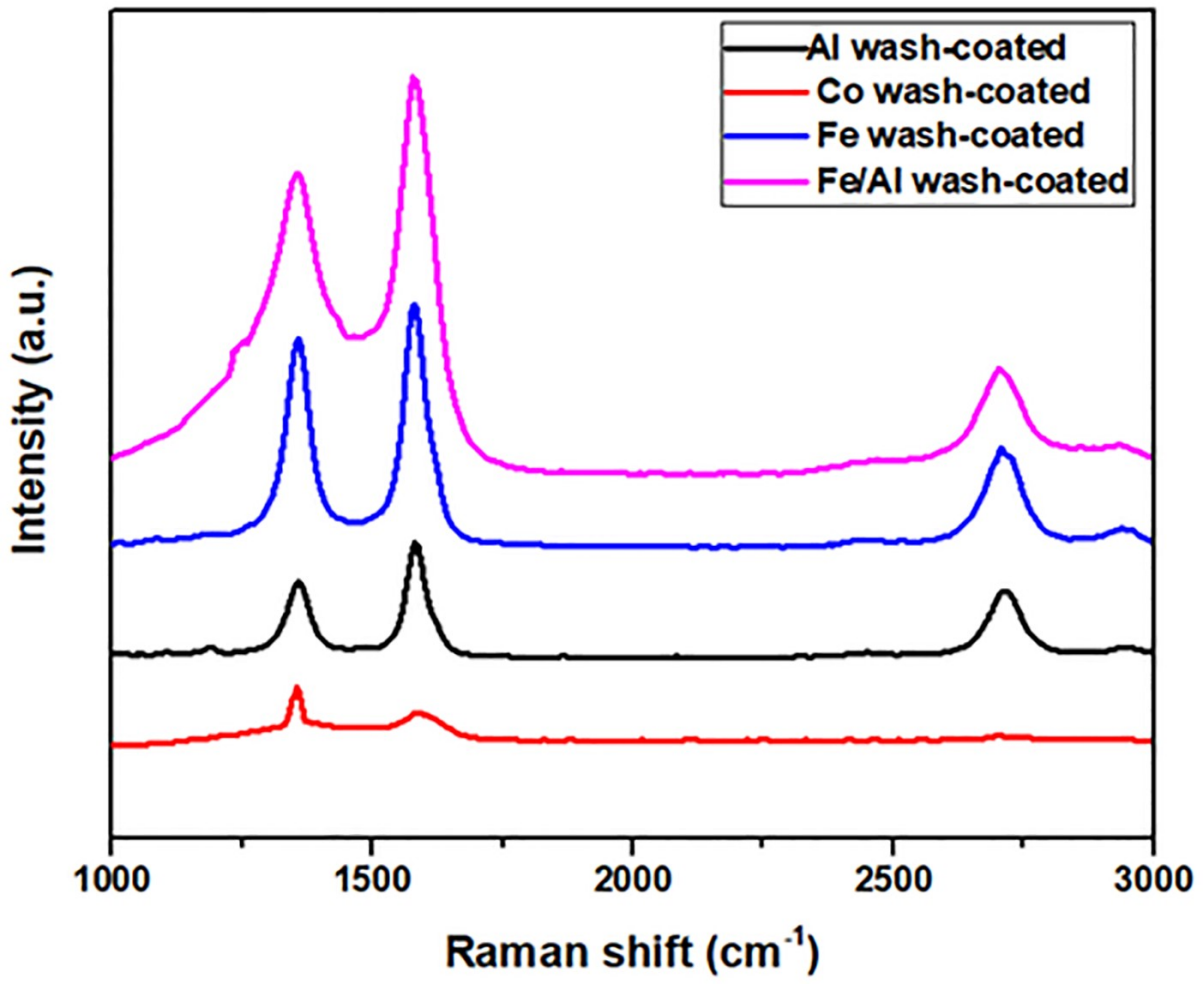
Raman spectra of different wash-coating samples; S1, S2, S3, and S4.

Raman spectra of the synthesized CNFs-coated monolith composite using various wash-coated materials are depicted in Fig 3. According to Raman spectra, All the synthesized sample had two vibration at ~1344 cm^-1^ (D band) and ~1578 cm^-1^ (G band) which were assigned to amorphous and graphitic structures of carbon, respectively. As it can be easily observed, that coated CNFs on sample S4 indicated two large and broad peaks at D and G peaks (FWHM of 85 cm^-1^ and 70 cm^-1^, respectively) as compared with the other sample. The FWHM of both D and G peaks are calculated for S1, S2, S3, and S4 to be 19 cm^-1^ and 29 cm^-1^, 47 cm^-1^ and 43 cm^-1^, 59 cm^-1^ and 54 cm^-1^, 85 cm^-1^ and 70 cm^-1^,respectively. Besides, the purity of the coated CNFs on sample S4 was confirmed with a low ID/IG ratio of 0.901, indicating the presence of low amount of graphitic impurities in comparison with other samples. The sample S2 showed the highest ratio of ID/IG (1.225) as compared with others, confirming the presence of impurities and amorphous carbon on the surface of active sites. The Raman results are highly in accordance with FE-SEM results.

The yield of coated CNFs on monolith using different wash-coated materials are shown in Table 1 as carbon yield were calculated using Eq (i). However, the S1 sample possessed a slightly higher CNF-coated yield as compared to S3 and S4 samples, CNFs were more deposited on monolith’s walls rather than in the monolith’s pores.

**Table 1:** S_BET_ and carbon yield of synthesized CNFs-coated monolith using different wash-coated metal oxides.

Sample	^a^S_BET_ (m^2^/g)	^b^CNF Yield (%)
Raw Monolith	1.8	2
Al Wash-coated	31.2	12
Fe Wash-coated	8.7	6
Al/Fe Wash-coated	35.7	10

^a^specific surface area

^b^yield of synthesized CNFs-coated monolith

As a result, the ICVD technique can be a promising technique to fabricate coated CNFs-coated monolith using wash-coated material (S4). Indeed, the ICVD method continuously introduced fresh catalyst which counteracted the effect of limited Iron staining, with more growth of CNFs occurring on the monolith substrates. This might be attributed to the presence of more quantity of Iron in the system which introduced in a controllable and uniform manner [15].

### 3.3 Characterization of CNFs-coated monolith

The textural, thermal and morphological properties of the synthesized CNFs-coated monolith were characterized by BET, TGA, and TEM, respectively.

**Fig 4:**
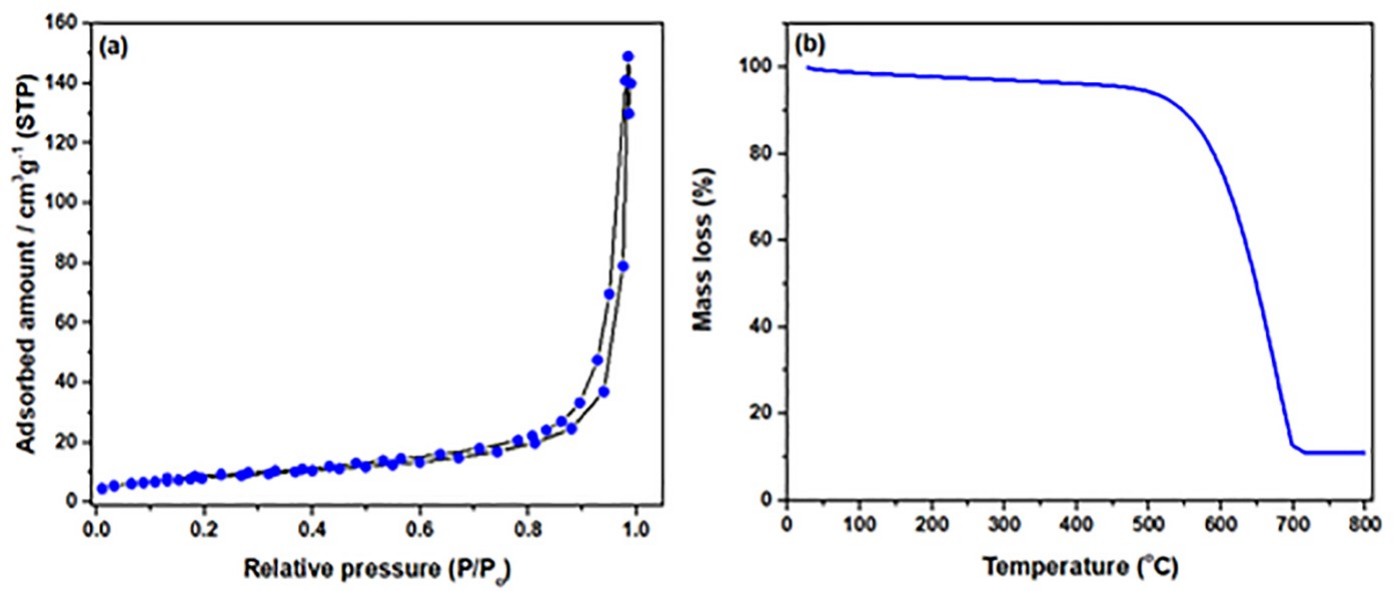
(a) N_2_ adsorption/isotherms of Al/Fe wash-coated monolith substrate; (b) The TGA of Al/Fe wash-coated monolith substrate.

Fig 4(a) displays the nitrogen adsorption/desorption isotherm of the fabricate CNFs-coated monolith. The synthesized material possessed a S_BET_ of 95.3 m^2^/g with the total pore volume of 0.021 cm^3^/g. The pore size distribution (based on BJH method) of the sample is about 1.8 nm microspores (<2nm) are available in the prepared sample. Results revealed microporous framework of the synthesized CNFs-coated monolith which were in line with previous reports [15]. The S_BET_ of coated CNFs on monolith using different wash-coated materials are shown in Table 1. Although, the S_BET_ of the wash-coated raw monolith with aluminum nitrate increased from 4 m^2^/g to 31.2 m^2^/g, the ICVD technique resulted in highly irregular coating of CNFs over the wash-coated monolith substrates due to disordered growth with random orientations. This disordered growth of CNFs over the wash-coated monolith substrates was previously reported in different studies [20–23].

The TGA was used to assess thermal decomposition performance of the fabricate coated CNFs-coated monolith as depicted in Fig 4(b). The mass-losses of 3% was observed at the temperature range of 26–150 °C, which was attributed to the decomposition of hydroxy-groups. The second weight-loss of 9% was detected at around 150–450 °C which was assigned to the decomposition of the carboxyl-groups (amorphous carbon) [24,25]. The third mass-losses at around 450 °C to 600 °C was corresponded to decomposition of the carbon nanofibers [26]. The major decomposition of the synthesized material took place at heating temperatures above 600 °C. The high stability of the synthesized CNFs-coated monolith may be also assigned to the strong attachment of the CNFs on the monolith substrate which disallowed the collapsing of the structure up to 600 °C. It disclosed that the coated CNFs over monolith substrate could possibly preserve the framework.

**Fig 5:**
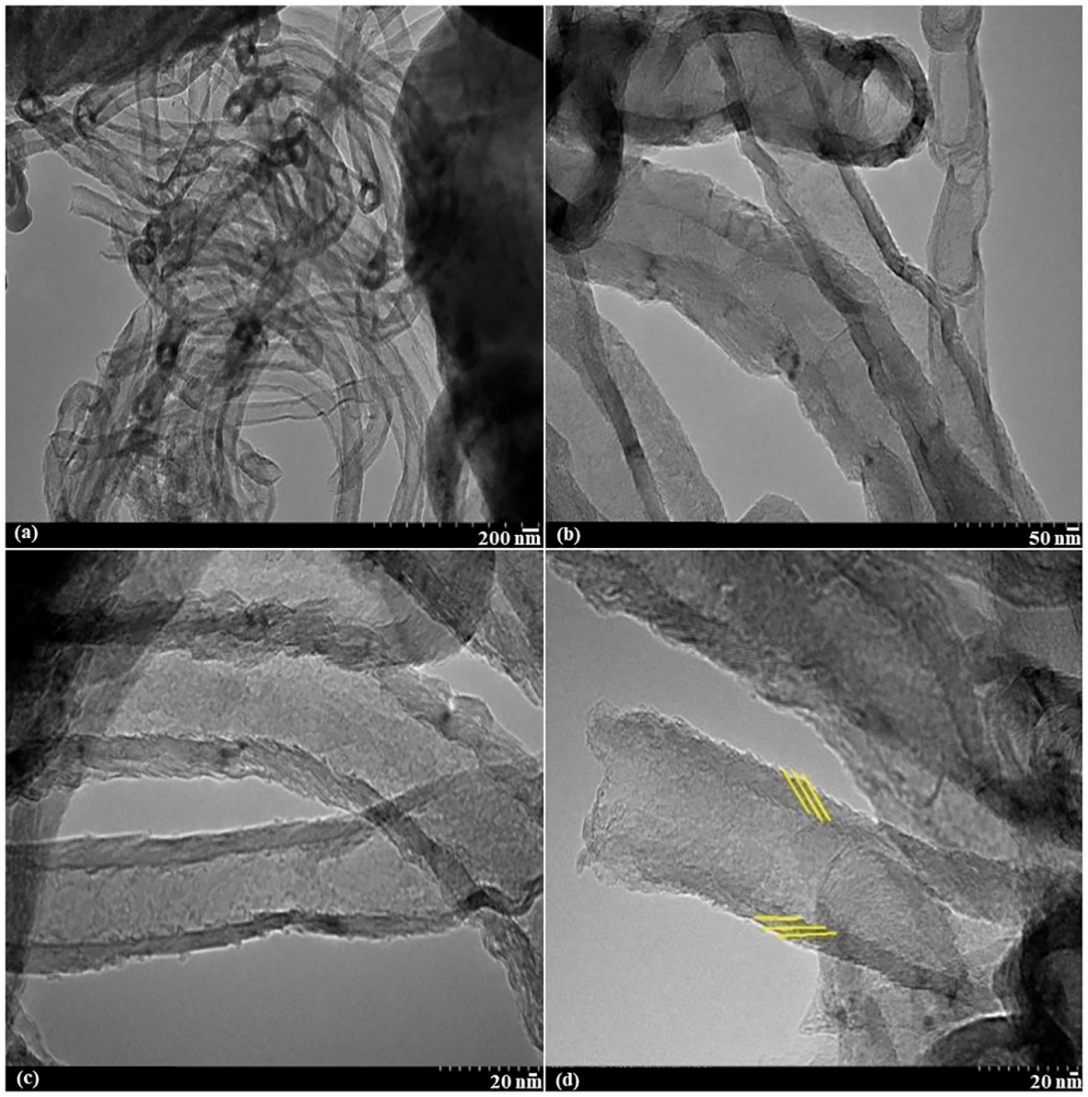
TEM images of CNFs using Al/Fe wash-coated monolith substrate.

Fig 5 depicts the TEM image of the fabricated CNFs-coated monolith substrate. As it can be evidently seen, the graphene planes were canted from the fiber axis, resulting in exposed edge planes on the interior and exterior surfaces of the fiber.

**Fig 6:**
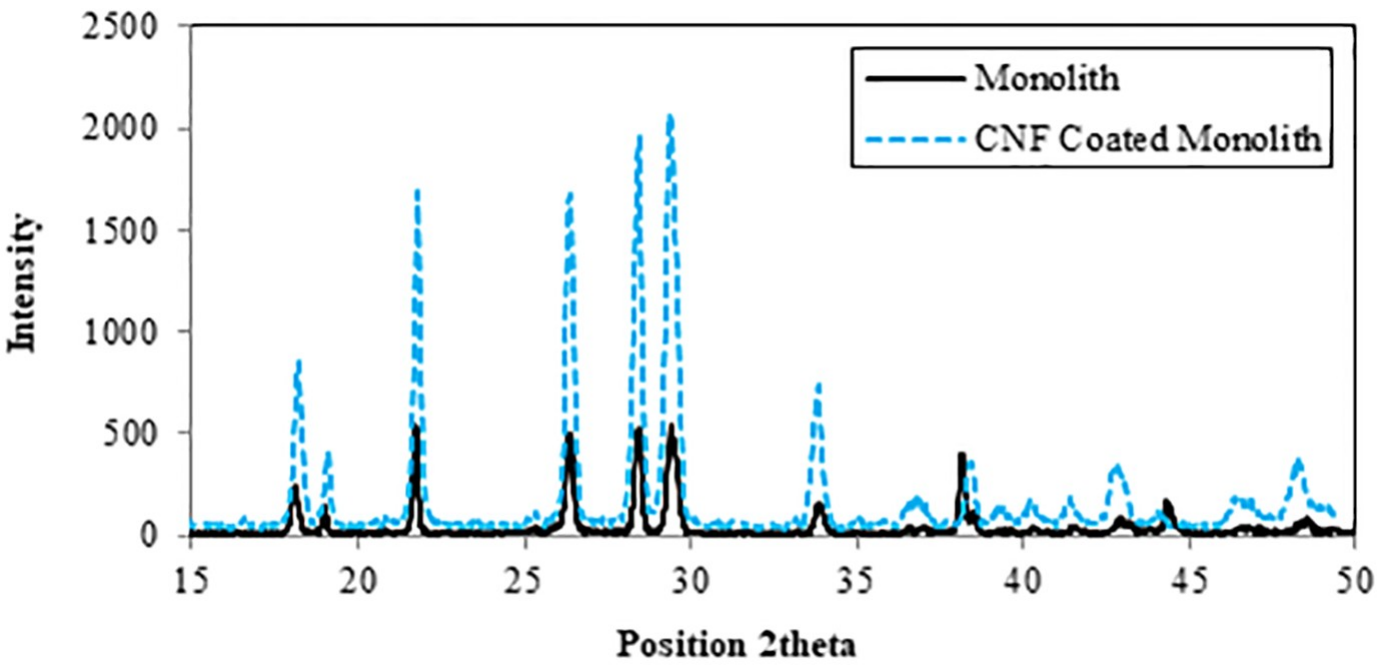
XRD pattern of CNFs using Al/Fe wash-coated monolith substrate.

Fig 6 represented the XRD pattern of CNF coated on monolith. As shown, there are sharp peak and strong intensity at about 2θ of 26.54, which was related to the (002) graphitic basal plane [27]. The XRD spectra includes several characteristic peaks around 42.54° and 44.44°, which would appear to suggest the presence of cementite an iron carbide with the chemical structure Fe_3_C. The other peaks in XRD pattern are clearly observed which were related to cordierite monolith [28].

## 4. Conclusions

In the present study, a sequence of experiments was performed to assess the different wash-coated metal oxides and catalyst promoters on the growth rate of CNFs over monolith substrates. Furthermore, the textural, structural, morphological characteristics and thermal resistance of the fabricated samples were characterized. The fabricated CNFs-coated monolith composite possessed a S_BET_ of 95.3 m^2^/g and total pore volume of 0.021 cm^3^/g. It is worthy to mention that the fabricated CNFs-coated monolith possessed extremely high graphitization degree with uniform and aligned distribution of CNFs on the surface of monolith substrate. The high stability of the synthesized CNFs-coated monolith was also confirmed which disclosed that the coated CNFs over monolith substrate could probably prohibited the collapsing of the structure up to 600 °C.
